# Predictive value of IGF-1/IGFBP-3 ratio for thyroid nodules in type 2 diabetic mellitus

**DOI:** 10.3389/fendo.2024.1444279

**Published:** 2024-10-09

**Authors:** Bingxin Liu, Yanjun Wang

**Affiliations:** Department of Endocrinology, The Second Hospital of Jilin University, Changchun, China

**Keywords:** type 2 diabetes mellitus, thyroid nodules, insulin resistance, insulin-like growth factor-1, insulin-like growth factor binding protein-3, IGF-1/IGFBP-3 ratio

## Abstract

**Aims:**

To explore the predictive value of the IGF-1/IGFBP-3 ratio for the presence of thyroid nodules in patients with type 2 diabetes mellitus (T2DM).

**Methods:**

This observational study prospectively enrolled patients with T2DM at the Second Hospital of Jilin University between May 2021 and January 2022. Thyroid nodule (TN) status was determined by ultrasonography. Receiver operating characteristic (ROC) curve analysis was performed to assess the predictive value of the serum IGF-1/IGFBP-3 molar ratio for TNs. Multivariable logistic regression analysis was conducted to identify risk factors for thyroid nodules in patients with T2DM.

**Results:**

A total of 122 patients (mean age ± standard deviation: 52.57 ± 11.71 years; 74 males) were enrolled. 37.7% (n=46) of patients did not have TNs, while 62.3% (n=76) had TNs. The duration of diabetes, age, and HDL-C level were significantly higher in the T2DM group with TNs compared to the group without TNs (all P < 0.05). The area under the ROC curve (AUC) for the combination of IGF-1, IGFBP-3, and the serum IGF-1/IGFBP-3 molar ratio in predicting TNs in T2DM patients was 0.619 (P < 0.001). Additionally, multivariable logistic regression analysis revealed that the duration of diabetes, age, fasting plasma glucose (FPG), fasting insulin (FINS), thyroid-stimulating hormone (TSH), IGF-1, and IGFBP-3 levels were independent risk factors for thyroid nodules, while the serum IGF-1/IGFBP-3 molar ratio level was an independent protective factor for thyroid nodules in patients with T2DM (all P < 0.05).

**Conclusion:**

The combination of IGF-1, IGFBP-3, and the serum IGF-1/IGFBP-3 molar ratio may have a better predictive value for TNs in T2DM patients than using any single marker alone. The duration of diabetes, age, FPG, FINS, TSH, IGF-1, IGFBP-3, and the serum IGF-1/IGFBP-3 molar ratio levels were independently associated with thyroid nodules in patients with T2DM.

## Introduction

Diabetes mellitus is an endocrine disorder characterized by chronic hyperglycemia resulting from defects in insulin secretion or action or both (1). It is a global health crisis with a rapidly increasing prevalence. According to a 2021 study, an estimated 536.6 million people aged 20-79 years worldwide have diabetes, and this number is projected to rise to 783.2 million by 2045. Type 2 diabetes (T2DM) is the most common form, accounting for approximately 90% of all diabetes cases. Several factors contribute to its growing prevalence, including obesity, unhealthy dietary habits, and genetic predisposition. The rise in type 2 diabetes poses a significant economic burden on healthcare systems and negatively impacts patient quality of life. Thyroid nodules are discrete, localized lesions within the thyroid gland that differ from the surrounding tissue. These nodules are quite common, with high-resolution ultrasound studies revealing a prevalence of 19% to 68% in the general population (2). Fortunately, the vast majority of thyroid nodules are benign and do not cause any clinical symptoms. However, similar to diabetes, the reported prevalence of thyroid nodules is on the rise worldwide. This trend might translate to increased healthcare utilization and associated patient costs.

Emerging evidence suggests a potential association between type 2 diabetes and thyroid nodules. Studies have demonstrated a significantly higher prevalence of thyroid nodules in patients with type 2 diabetes compared to those without. For instance, one study reported a prevalence of 60% in type 2 diabetic patients, compared to 43% in non-type 2 diabetic patients (3). Several mechanisms may underlie this connection. One hypothesis suggests that compensatory insulin resistance, a hallmark of type 2 diabetes, leads to elevated serum insulin levels, which may be linked to thyroid nodule formation (4). Additionally, research suggests that increased systemic inflammation, often observed in type 2 diabetes, might also play a role in developing thyroid nodules in this population (5).

The insulin-like growth factor (IGF) family comprises a group of peptide hormones with high structural similarity to insulin. IGFs are essential for various physiological processes, including cell proliferation, apoptosis, protein synthesis, and regulation of carbohydrate, fat, and bone metabolism (6). Notably, IGF-1 and IGFBP-3 have been implicated in developing thyroid nodules in patients with type 2 diabetes mellitus. In T2DM, a hallmark feature is insulin resistance. This resistance may directly activate the proliferation pathway through insulin or IGF-1, potentially influencing the expression of thyroid-related genes and promoting thyroid cell proliferation and differentiation (7). Studies suggest that IGF-1 and IGFBP-3 influence peripheral insulin action through various mechanisms. IGFBP-3 also exerts independent physiological effects. It can inhibit insulin action through both IGF-1-dependent and IGF-1-independent pathways, potentially contributing to the development of metabolic syndrome (8). Importantly, in circulation, most IGF-1 binds to IGFBP-3 and other proteins, forming a large complex (approximately 150 kDa). This complex limits the free IGF-1 available for cellular uptake and utilization (6). Therefore, a reliable marker is needed to estimate the biologically active IGF-1 fraction. The molar ratio of serum IGF-1 to IGFBP-3 has been proposed to assess IGF-1 bioactivity (9). It is calculated by dividing the serum IGF-1 concentration (ng/mL) by 0.13 and then dividing this value by the serum IGFBP-3 concentration (ng/mL) divided by 0.035 (10). However, serum IGF-1 and IGFBP-3 levels may not always change proportionally. For example, a study showed that on rGH treatment, the IGF-1-dependent increase in IGFBP-3 concentration occurs in two phases: an initial linear phase, followed by a saturation phase in which IGFBP-3 concentrations reach a plateau, despite further increases in IGF-1 concentration. This would account for the increase in IGF-1/IGFBP-3 ratio at high doses of rGH (11). In such scenarios, the IGF-1/IGFBP-3 molar ratio can serve as a surrogate marker for both IGF-1 and IGFBP-3 activity. Additionally, this ratio has proven valuable in research on other diseases (12). With current guidelines generally advising against routine ultrasound screening for thyroid nodules in the general population, a combined predictive approach using IGF-1, IGFBP-3, and the IGF-1/IGFBP-3 ratio might hold promise for preventing thyroid nodules in T2DM patients.

The present study investigated the potential of the IGF-1/IGFBP-3 molar ratio as a predictor of thyroid nodules in patients diagnosed with type 2 diabetes mellitus.

## Methods

### Study design and participants

This observational study enrolled a total of 122 patients with T2DM at the Second Hospital of Jilin University between May 2021 and January 2022. The study protocol was approved by the Ethics Committee of the Second Hospital of Jilin University (approval number: 2024-078). All data were anonymized. As the study involved minimal risk and collected anonymized data, the requirement for informed consent was waived by the Ethics Committee.

This study included participants between 18 and 80 years old who fulfilled the 2020 Chinese guidelines for diagnosing Type 2 Diabetes Mellitus (13). To ensure a focused and generalizable study population, we excluded patients with (1) type 1 diabetes or other atypical diabetes types, (2) acute or severe chronic complications from diabetes, (3) severe liver, kidney, heart, or other organ diseases, (4) pre-existing thyroid disease or use of medications affecting glucose metabolism or thyroid function, (5) blood system or immune system disorders, and (6) any serious infectious disease or malignancy.

### Data collection and definitions

Demographic and clinical data, including sex, age, duration of diabetes mellitus (course of disease), weight, and height, were collected. Body mass index (BMI) was calculated using the formula BMI = weight (kg)/height² (m²). Additionally, general biochemical biomarkers were assessed. Glycated hemoglobin A1c (HbA1c) was measured using an automated hemoglobin analyzer (HLC-723G8). An automated biochemical analyzer (UniCel DxC 800 Synchron) was used to determine fasting blood glucose (FPG), fasting serum insulin (FINS), fasting C-peptide (FC-P), triglycerides (TG), total cholesterol (TC), low-density lipoprotein cholesterol (LDL-C), high-density lipoprotein cholesterol (HDL-C), thyroid peroxidase antibody (TPOAb), thyroglobulin antibody (TgAb), free triiodothyronine (FT3), free thyroxine (FT4), thyroid stimulating hormone (TSH), and uric acid (UA). Urine microalbumin (UMA) and urinary albumin-to-creatinine ratio (UACR) were measured using a protein analyzer (IMMAGE800). Finally, the homeostasis model assessment of insulin resistance (HOMA-IR) was calculated using the formula: HOMA-IR=FPG (mmol/L) × FINS (U/mL)/22.5.

All patients underwent thyroid ultrasound examinations performed by qualified ultrasound physicians within the same ultrasound medicine department. Standardized examinations were ensured using the same ultrasound frequency instrument (PHILIPEPIQ7, 5-18MHz; Philips) for all procedures. Following the diagnostic criteria outlined in the 2020 Chinese guidelines for malignant risk stratification of thyroid nodules (C-TIRADS), patients with type 2 diabetes were categorized into two groups: a simple type 2 diabetes group and a type 2 diabetes with thyroid nodules group.

### Methods for the detection of IGF-1, IGFBP-3 and IGF-1/IGFBP-3 molar ratio

Serum concentrations of IGF-1 and IGFBP-3 were measured using an immunochemiluminometric assay (ICMA) on a Siemens 2000XPI platform. The molar ratio of serum IGF-1 to IGFBP-3 was calculated using the following formula: molar ratio = [IGF-1 (ng/mL)×0.13]/[IGFBP-3 (ng/mL)×0.035] (10).

### Statistical analysis

Data analysis was performed using the statistical software SPSS version 29.0 (IBM Corp., Armonk, NY, USA). Continuous data with normal distribution were presented as mean ± standard deviation (SD) and analyzed using the Student’s t-test. Conversely, continuous data with skewed distribution were expressed as median (interquartile range) and analyzed using the Mann-Whitney U test. Categorical data were presented as percentages (%) and analyzed using the chi-square test. Pearson correlation analysis was used to assess correlations between normally distributed continuous variables. Spearman correlation analysis was employed to evaluate correlations between categorical and continuous data with skewed distribution. The receiver operating characteristic (ROC) curve was generated using GraphPad Prism version 10.0 (GraphPad Software Inc., San Diego, CA, USA). The area under the curve (AUC) was calculated to assess the predictive value of serum IGF-1, IGFBP-3, and the IGF-1/IGFBP-3 molar ratio for thyroid nodules in patients with T2DM. Multivariable logistic regression analysis was conducted to identify risk factors for thyroid nodules in patients with T2DM. A two-tailed p-value of less than 0.05 was considered statistically significant.

## Results

A total of 122 patients with T2DM were enrolled in this study. The mean age was 52.6 ± 11.7 years, with 74 males and 48 females. Between the two groups (simple T2DM <i>vs</i>. T2DM with thyroid nodules), there were statistically significant differences (all P < 0.05) in the duration of type 2 diabetes (course of disease), age, and HDL-C levels. Specifically, the T2DM with thyroid nodules group had a longer duration of diabetes, higher age, and higher HDL-C levels than the simple T2DM group. No significant differences were observed in other clinical parameters between the two groups (all P > 0.05) (**Table 1**).

**Table 1:** Characteristics of the patients.

Variables	Control group (n=46)	Thyroid nodule group (n=76)	P values
Course of disease (years)	4.50 (9.06)	6.50 (10.75)	**0.021**
Sex (%)			
Male	31	43	0.236
Female	15	33	
Age (years)	48.67±10.63	54.93±11.76	**0.004**
Height (cm)	168.93±7.56	167.25±8.71	0.279
Weight (kg)	75.27±12.85	73.65±13.93	0.515
BMI (kg/m^2^)	26.30±3.41	26.26±3.63	0.95
HbA1C (%)	8.50 (2.40)	7.90 (2.30)	0.389
FPG (mmol/L)	9.20 (4.73)	9.12 (3.89)	0.949
FINS (mU/L)	15.28 (11.07)	15.69 (14.49)	0.59
FC-P (ng/mL)	1.53 (1.20)	1.68 (1.23)	0.947
IGF-1 (ng/mL)	151.50 (57.25)	142.00 (75.50)	0.618
IGFBP-3 (μg/mL)	5.20±1.49	5.21±1.60	0.973
TPOAb (U/mL)			
<9.00	23	39	0.888
>=9.00	23	37	
TgAb (U/mL)			
<10	26	43	0.995
>=10	20	44	
FT3 (pmol/L)	4.51±0.79	4.40±0.75	0.429
FT4 (pmol/L)	17.78±2.95	16.89±2.94	0.106
TSH (mU/L)	1.88 (1.19)	2.24 (1.69)	0.161
TG (mmol/L)	1.90 (1.03)	1.87 (1.59)	0.419
TC (mmol/L)	5.68 (1.60)	5.57 (1.71)	0.759
LDL-C (mmol/L)	3.09±0.94	3.07±1.10	0.926
HDL-C (mmol/L)	1.06±0.26	1.17±0.28	**0.033**
MA (mg/L)	10.00 (20.00)	10.00 (20.00)	0.856
UA (umol/L)	328.50 (126.00)	330.00 (111.00)	0.69
UACR (mg/g)			
<31	37	58	0.595
>=31	9	18	
HOMA-IR	6.14 (5.81)	6.69 (6.13)	0.476
IGF-1/IGFBP-3 ratio	0.11 (0.05)	0.11 (0.04)	0.866

The bold means that the P value is less than 0.05.

**Figure 1:**
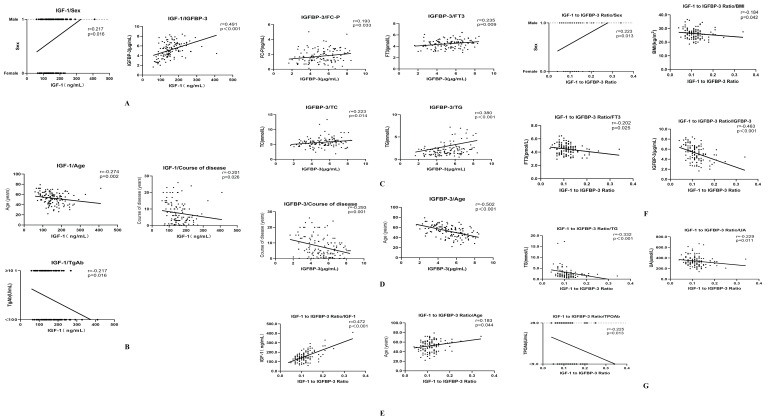
**(A)** Correlation analysis of IGF-1 with sex and IGFBP-3. **(B)** Correlation analysis of IGF-1 with course of disease, age and TgAb. **(C)** Correlation analysis of IGFBP-3 with Fasting c-peptide (FC-P), Free triiodothyronine (FT3), Total cholesterol (TC) and Triglycerides (TG). **(D)** Correlation analysis of IGFBP-3 with the course of disease and age. **(E)** Correlation analysis of IGF-1/IGFBP-3 ratio with IGF-1 and age. **(F)** Correlation analysis of IGF-1/IGFBP-3 ratio with sex, BMI, FT3 and IGFBP-3. **(G)** Correlation analysis of IGF-1/IGFBP-3 ratio with TG, Uric acid (UA) and Thyroid peroxidase antibody (TPOAb).

Serum IGF-1 levels exhibited positive correlations with sex and IGFBP-3, while negative correlations were observed with duration of disease, age, and TgAb (anti-thyroglobulin antibody) levels. (r=0.217, r=0.491, r=-0.201;r=-0.274;r=-0.217, respectively; all P<0.05) (**Figures 1A, B**, **Table 2**). IGFBP-3 levels were positively correlated with FC-P, FT3, TC, and TG, while negatively correlated with the course of disease and age. (r=0.193, r=0.235, r=0.223, r=0.380, r=-20.293, r=-0.502, respectively; all P<0.05) (**Figures 1C, D**, **Table 2**). The IGF-1/IGFBP-3 molar ratio demonstrated positive correlations with IGF-1, age, and sex. Conversely, negative correlations were found with BMI, FT3, IGFBP-3, TG, UA, and TPOAb levels. (r=0.472, r=0.183, r=0.223, r=-0.184, r=-0.202, r=-463, r=-0.332, r=-0.229, r=-0.225, all P < 0.05) (**Figures 1E–G**, **Table 2**).

**Table 2:** Correlation analysis of IGF-1,IGFBP-3,IGF-1/IGFBP-3 ratio and clinical biomarkers.

Variables	IGF-1		IGFBP-3		IGF-1/IGFBP-3 ratio	
	r/r_s_	P	r/r_s_	P	r/r_s_	P
Course of disease (years)	-0.201	**0.026**	-0.293	**0.001**	0.076	0.407
Sex	0.217	**0.016**	0.028	0.761	0.223	**0.013**
Age (years)	-0.274	**0.002**	-0.502	**<0.001**	0.183	**0.044**
BMI(kg/m2)	-0.053	0.561	0.16	0.079	-0.184	**0.042**
HbA1C (%)	-0.094	0.304	0.035	0.703	0.068	0.455
FPG(mmol/L)	-0.093	0.307	0.114	0.211	0.165	0.07
FINS(mU/L)	0.038	0.676	-0.036	0.693	0.057	0.536
FC-P(ng/mL)	0.127	0.162	0.193	**0.033**	0.082	0.37
TPOAb(U/mL)	-0.074	0.415	0.08	0.383	-0.225	**0.013**
TgAb(U/mL)	-0.217	**0.016**	-0.114	0.211	-0.116	0.202
FT3(pmol/L)	0.045	0.62	0.235	**0.009**	-0.202	**0.025**
FT4(pmol/L)	-0.005	0.956	0.046	0.612	-0.07	0.442
TSH(mU/L)	-0.12	0.188	-0.097	0.287	-0.017	0.852
TG(mmol/L)	0.052	0.57	0.38	**<0.001**	-0.332	**<0.001**
TC(mmol/L)	0.094	0.301	0.223	**0.014**	-0.17	0.062
LDL-C(mmol/L)	0.071	0.438	0.019	0.832	0.015	0.867
HDL-C(mmol/L)	-0.044	0.627	-0.072	0.432	0.002	0.981
MA(mg/L)	0.063	0.49	0.157	0.084	-0.139	0.127
UA(umol/L)	0.063	0.489	0.17	0.06	-0.229	**0.011**
UACR(mg/g)	0.108	0.237	0.043	0.635	0.022	0.809
HOMA-IR	-0.022	0.811	0.028	0.762	-0.049	0.595
IGF-1(ng/mL)			0.491	**<0.001**	0.472	**<0.001**
IGFBP-3(μg/mL)					-0.463	**<0.001**

The bold means that the P value is less than 0.05.

**Figure 2:**
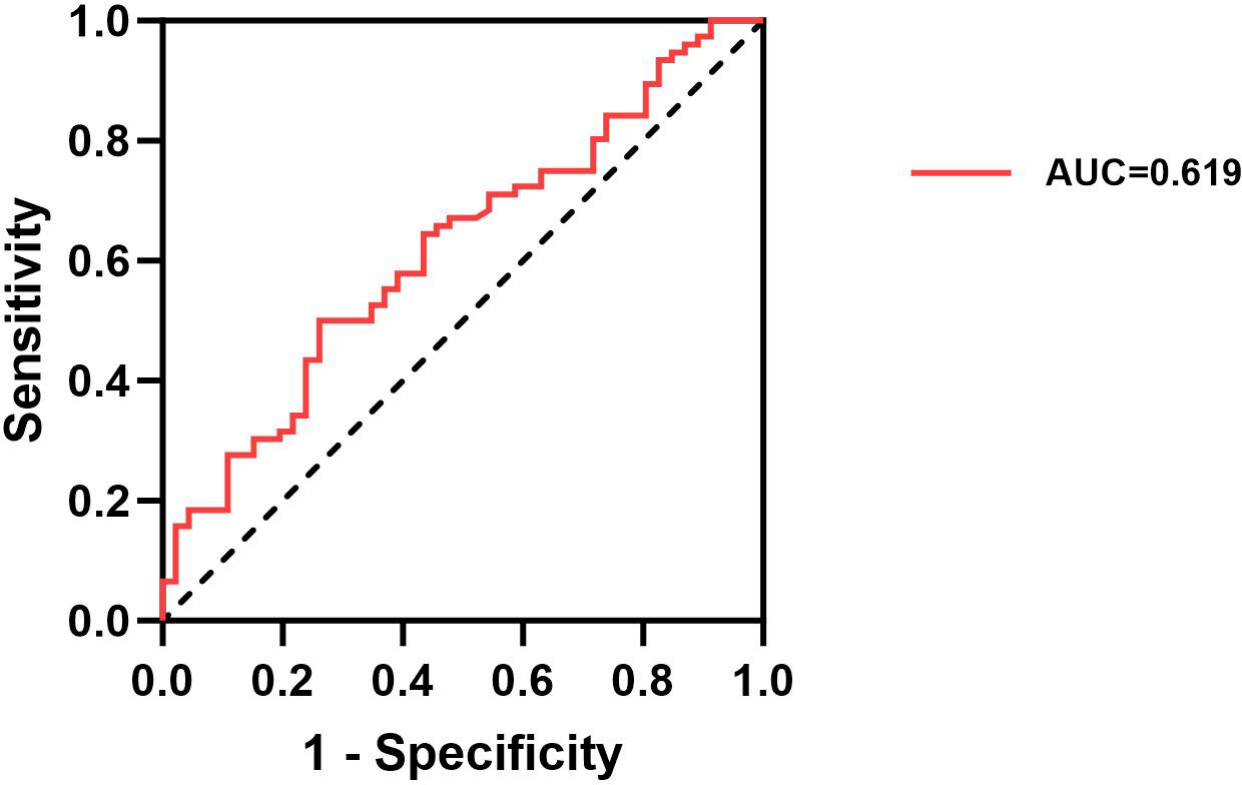
ROC curve of the combination of IGF-1, IGFBP-3, IGF-1/IGFBP-3 ratio for TNs in T2DM.

The ROC curve analysis evaluated the potential of serum IGF-1, IGFBP-3, and the IGF-1/IGFBP-3 molar ratio as predictors of thyroid nodules in patients with T2DM. The results indicated that IGF-1, IGFBP-3, and the IGF-1/IGFBP-3 ratio individually exhibited an uncertain predictive value for thyroid nodules in patients with T2DM (AUC=0.527, AUC=0.501, AUC=0.504, P>0.05) (**Table 3**). In contrast, the AUC for the combined model incorporating IGF-1, IGFBP-3, and the IGF-1/IGFBP-3 molar ratio demonstrated a significant association with the presence of thyroid nodules in T2DM patients (AUC = 0.619, P < 0.001) (**Figure 2**, **Table 3**).

**Table 3:** Predictive value of IGF-1, IGFBP-3, IGF-1/IGFBP-3 ratio for TNs in T2DM.

Variables	AUC	95% CI	Sensitivity (%)	Specificity (%)	YI	P
IGF-1	0.527	(0.424, 0.630)	0.184	0.978	0.163	0.618
IGFBP-3	0.501	(0.395, 0.606)	0.553	0.522	0.074	0.990
IGF-1/IGFBP-3 ratio	0.504	(0.393,0.615)	0.908	0.217	0.125	0.943
Combination biomarkers	0.619	(0.518, 0.721)	0.500	0.739	0.239	**0.027**

The bold means that the P value is less than 0.05.

Additionally, univariable logistic regression analysis showed that sex, age and HDL-C were potential risk factors for thyroid nodules in type 2 diabetic mellitus. (all P<0.05) (**Table 4**). Furthermore, multivariable logistic regression analysis suggested that age (OR=1.063, 95%CI 1.019-1.109, P=0.005), FPG (OR=1.508, 95%CI 1.066-2.134, P=0.020) and FINS (OR=1.213, 95%CI 1.010-1.455, P=0.038) were independent risk factors for thyroid nodules in type 2 diabetic mellitus, while IGF-1/IGFBP-3 ratio (OR<0.001, 95%CI <0.001-0.748, P=0.044) was inversely independent protective factors for thyroid nodules in type 2 diabetic mellitus (**Table 5**). Age (OR=1.082, 95%CI 1.030-1.136, P=0.002), FPG (OR=1.493, 95%CI 1.047-2.128, P=0.027), TSH (OR=1.493, 95%CI 1.002-2.225, P=0.049) and IGFBP-3 (OR=1.414, 95%CI 1.019-1.963, P=0.038) were independent risk factors for thyroid nodules in type 2 diabetic mellitus (**Table 6**). Age (OR=1.094, 95%CI 1.038-1.153, P<0.001), FPG (OR=1.516, 95%CI 1.067-2.156, P=0.020) and IGF-1 (OR=1.040, 95%CI 1.003-1.079, P=0.033) were independent risk factors for thyroid nodules in type 2 diabetic mellitus, while IGF-1/IGFBP-3 ratio (OR<0.001, 95%CI <0.001-<0.001, P=0.020) was inversely independent protective factors for thyroid nodules in type 2 diabetic mellitus (**Table 7**).

**Table 4:** Univariable logistic analysis of the influence of TNs in T2DM.

Variables	B	SE	Walds	P	OR	95% CI
Course of disease (years)	-0.461	0.39	1.395	0.238	0.63	(0.293,1.355)
Sex	0.076	0.032	5.507	**0.019**	1.079	(1.013,1.150)
Age (years)	0.048	0.017	7.786	**0.005**	1.049	(1.014,1.085)
BMI (kg/m2)	-0.003	0.053	0.004	0.949	0.997	(0.898,1.106)
HbA1C (%)	-0.069	0.116	0.355	0.551	0.933	(0.744,1.171)
FPG (mmol/L)	0.007	0.061	0.012	0.911	1.007	(0.893,1.136)
FINS (mU/L)	0.015	0.016	0.926	0.336	1.015	(0.985,1.047)
FC-P (ng/mL)	-0.014	0.21	0.005	0.946	0.986	(0.653,1.489)
TPOAb (U/mL)	0.053	0.374	0.02	0.888	1.054	(0.507,2.192)
TgAb (U/mL)	0.002	0.377	<0.001	0.995	1.002	(0.479,2.098)
FT3 (pmol/L)	-0.196	0.247	0.635	0.425	0.822	(0.507,1.332)
FT4 (pmol/L)	-0.106	0.066	2.583	0.108	0.9	(0.791,1.023)
TSH (mU/L)	0.278	0.16	3.011	0.083	1.321	(0.965,1.809)
TG (mmol/L)	-0.049	0.051	0.946	0.331	0.952	(0.861,1.052)
TC (mmol/L)	0.012	0.118	0.011	0.918	1.012	(0.804,1.274)
LDL-C (mmol/L)	-0.017	0.18	0.009	0.925	0.983	(0.691,1.400)
HDL-C (mmol/L)	1.555	0.742	4.395	**0.036**	4.733	(1.107,20.247)
MA (mg/L)	-0.001	0.004	0.026	0.871	0.999	(0.992,1.007)
UA (umol/L)	-0.002	0.002	0.839	0.36	0.998	(0.994,1.002)
UACR (mg/g)	-0.244	0.459	0.281	0.596	0.784	(0.319,1.928)
HOMA-IR	0.005	0.033	0.026	0.873	1.005	(0.943,1.072)
IGF-1 (ng/mL)	<0.001	0.003	<0.001	0.987	1	(0.993,1.007)
IGFBP-3 (μg/mL)	0.004	0.121	0.001	0.972	1.004	(0.792,1.272)
IGF-1/IGFBP-3 ratio	-2.744	4.487	0.374	0.541	0.064	(<0.001.424.265)

The bold means that the P value is less than 0.05.

**Table 5:** Multivariable logistic analysis of the influence of TNs in T2DM.

Variables	B	SE	Walds	P	OR	95% CI
Sex	0.589	0.5	1.387	0.239	1.803	(0.676,4.806)
Age (years)	0.061	0.022	8.043	**0.005**	1.063	(1.019,1.109)
FPG (mmol/L)	0.411	0.177	5.379	**0.02**	1.508	(1.066,2.134)
FINS (mU/L)	0.193	0.093	4.283	**0.038**	1.213	(1.010,1.455)
FT4 (pmol/L)	-0.077	0.082	0.889	0.346	0.926	(0.789,1.087)
TSH (mU/L)	0.338	0.202	2.796	0.094	1.403	(0.943,2.085)
HDL-C (mmol/L)	1.549	0.879	3.105	0.078	4.708	(0.840,26.370)
HOMA-IR	-0.391	0.222	3.114	0.078	0.676	(0.438,1.044)
IGF-1/IGFBP-3 ratio	-11.408	5.672	4.045	**0.044**	<0.001	(<0.001,0.748)

**Table 6:** Multivariable logistic analysis of the influence of TNs in T2DM.

Variables	B	SE	Walds	P	OR	95% CI
Sex	0.464	0.486	0.91	0.34	1.59	(0.613,4.121)
Age (years)	0.079	0.025	9.913	**0.002**	1.082	(1.030,1.136)
FPG(mmol/L)	0.401	0.181	4.913	**0.027**	1.493	(1.047,2.128)
FINS(mU/L)	0.182	0.096	3.588	0.058	1.2	(0.994,1.449)
FT4(pmol/L)	-0.074	0.081	0.827	0.363	0.929	(0.793,1.089)
TSH(mU/L)	0.401	0.204	3.879	**0.049**	1.493	(1.002,2.225)
HDL-C(mmol/L)	1.413	0.932	2.296	0.13	4.107	(0.661,25.537)
HOMA-IR	-0.362	0.228	2.532	0.112	0.696	(0.445,1.088)
IGFBP-3	0.347	0.167	4.297	**0.038**	1.414	(1.019,1.963)

**Table 7:** Multivariable logistic analysis of the influence of TNs in T2DM.

Variables	B	SE	Wald	P	OR	95% CI
Sex	0.693	0.527	1.729	0.189	2.001	(0.712,5.624)
Age (years)	0.09	0.027	11.08	**<0.001**	1.094	(1.038,1.153)
FPG(mmol/L)	0.416	0.18	5.378	**0.02**	1.516	(1.067,2.156)
FINS(mU/L)	0.176	0.095	3.419	0.064	1.193	(0.989,1.437)
FT4(pmol/L)	-0.083	0.085	0.958	0.328	0.92	(0.780,1.087)
TSH(mU/L)	0.416	0.221	3.559	0.059	1.516	(0.984,2.336)
HDL-C(mmol/L)	1.238	0.935	1.754	0.185	3.448	(0.552,21.537)
HOMA-IR	-0.334	0.22	2.3	0.129	0.716	(0.465,1.103)
IGF-1	0.04	0.018	4.569	**0.033**	1.04	(1.003,1.079)
IGFBP-3	-0.832	0.53	2.465	0.116	0.435	(0.154,1.229)
IGF-1/IGFBP-3 ratio	-59.402	25.567	5.398	**0.02**	<0.001	(<0.001, <0.001)

## Discussion

This study suggests that a combined model incorporating serum IGF-1, IGFBP-3, and the IGF-1/IGFBP-3 molar ratio may improve TN prediction for TNs in patients with T2DM. Additionally, our findings indicate independent associations between TNs and factors, including disease duration, age, FPG, FINS, TSH, IGF-1, IGFBP-3, and the IGF-1/IGFBP-3 ratio. These results hold promise for developing a predictive model for TNs in T2DM patients.

Insulin resistance is a prevalent clinical condition, particularly in T2DM. Existing retrospective studies support a significant link between insulin resistance and thyroid nodules (14). Junik et al. (15) reported a higher prevalence of thyroid nodules in patients with T2DM compared to non-diabetic individuals without insulin resistance. Also, in type 2 diabetes mellitus, the levels of IGF-1, IGFBP-3 and IGFBP-1 may be extremely variable (16). Characterized by hyperglycemia and elevated insulin levels, insulin resistance promotes the interaction between insulin and insulin-like growth factor binding proteins (IGFBPs), increasing free IGF-1 levels (17), This, in turn, may influence cellular regulation and proliferation within thyroid tissue (18). Our finding that FPG and FINS are risk factors for TNs in T2DM patients further supports the potential association between insulin resistance and TN pathogenesis. Notably, another study demonstrated higher insulin resistance in the thyroid nodule group compared to the control group, further supporting the concept of insulin resistance as a risk factor for TNs in T2DM patients (19).

Our findings suggest a close link between the IGF system and TN development in patients with T2DM. Multivariable logistic regression analysis identified independent risk factors for TNs, including duration of disease, age, FPG, FINS, TSH, and both IGF-1 and IGFBP-3 levels. Interestingly, the serum IGF-1/IGFBP-3 molar ratio emerged as an independent protective factor for TNs in this population. Existing research supports the role of IGF-1 in stimulating protein and DNA synthesis within thyroid cells (20). It acts synergistically with TSH through an autocrine mechanism to promote thyroid cell proliferation, potentially contributing to TN occurrence and development (21). In an *in vitro* study, the mitogenic effect of IGF-1 on thyroid cells was also confirmed (22). The insulin/IGF-1 pathway is further implicated by studies suggesting that insulin resistance may influence TN formation (23). Hard et al. observed in patients with type 2 diabetes (24) observed that hyperinsulinemia in patients with type 2 diabetes can block apoptosis and promote cell proliferation by stimulating insulin and IGF-1 pathways. The high degree of homology between insulin and IGF-1 receptors allows insulin to mimic IGF-1 and bind to its receptor, subsequently activating mitogen-activated protein kinase (MAPK) and phosphatidylinositol 3-kinase (PI3K) pathways, ultimately promoting thyroid cell proliferation (25). IGFBP-3 plays a counterbalancing role by binding to IGF-1 and reducing its bioactive level, indirectly regulating thyroid cell proliferation. Additionally, IGFBP-3 activates phosphotyrosine phosphatase, downregulating the IGF-1 signaling pathway, further influencing cell proliferation (26). Notably, our multivariable analysis, considering total rather than free IGF-1 levels, suggests a potentially stronger influence of IGFBP-3 compared to IGF-1 on TN development in T2DM. This is further supported by the observed positive correlation between IGFBP-3 and FC-P levels in this study, hinting at an association between IGFBP-3 and TNs that may be independent of IGF-1. Collectively, these findings highlight the central role of the IGF-1/IGFBP-3 molar ratio in the pathogenesis of TNs in patients with T2DM.

Current trends in clinical guidelines suggest a decreasing emphasis on routine ultrasound screening for thyroid nodules in the general population. In this context, the possibility of screening for TNs in patients with T2DM using a simple blood test holds significant appeal. As demonstrated by the ROC curve analysis, the AUC for the combined model incorporating serum IGF-1, IGFBP-3, and the IGF-1/IGFBP-3 molar ratio was 0.619. This finding indicates that combining these three biomarkers offers a more reliable predictive value for TNs in T2DM patients than using any single marker alone.

Several limitations in this study should be acknowledged. First, the single-center design resulted in a relatively small sample size, potentially limiting the generalizability of our findings to broader populations. Second, the cross-sectional nature of the study precludes establishing causal relationships between the investigated factors and thyroid nodules. Future prospective observational studies are warranted to address this limitation. Finally, the current study lacked the ability to measure free IGF-1 levels directly. Since the serum IGF-1/IGFBP-3 molar ratio is influenced by various physiological and pathophysiological factors, relying on total IGF-1 measurements may introduce some degree of error (27).

This study suggests that a combined model incorporating serum IGF-1, IGF-1/IGFBP-3 molar ratio, and IGFBP-3 may offer a more robust predictive value for TNs in patients with T2DM compared to using individual biomarkers. Additionally, our findings identified independent associations between TNs and factors, including duration of disease, age, FPG, FINS, TSH, and both IGF-1 and IGFBP-3 levels. However, the proposed prediction model based on the IGF-1/IGFBP-3 molar ratio for TNs in T2DM patients requires further validation through multicenter studies with larger sample sizes.

## Data Availability

The original contributions presented in the study are included in the article/supplementary material, further inquiries can be directed to the corresponding author.
